# Follicular Fluid Vanin-1 Levels in Patients Undergoing Ivf: A Preliminary Study

**DOI:** 10.3390/antiox14020133

**Published:** 2025-01-23

**Authors:** Ákos Várnagy, Péter Mauchart, Gábor Nagy, József Bódis, Endre Sulyok

**Affiliations:** 1 National Laboratory on Human Reproduction, University of Pécs, H-7624 Pécs, Hungary; varnagy.akos@pte.hu (Á.V.); bodis.jozsef@pte.hu (J.B.); esulyok@t-online.hu (E.S.); 2 HUN-REN–PTE Human Reproduction Research Group, H-7624 Pécs, Hungary; 3 Department of Obstetrics and Gynecology, School of Medicine, University of Pécs, H-7624 Pécs, Hungary; 4 Department of Laboratory Medicine, Faculty of Medicine, University of Debrecen, H-4032 Debrecen, Hungary; nagy.gabor@med.unideb.hu; 5 Doctoral School of Health Sciences, Faculty of Health Sciences, University of Pécs, H-7624 Pécs, Hungary

**Keywords:** vanin-1, follicular fluid, in vitro fertilization, pregnancy, oxidative stress

## Abstract

This preliminary study was designed to determine follicular fluid (FF) vanin-1 levels, to assess their relation to serum vanin-1 and to reveal their potential to predict the outcome of in vitro fertilization (IVF). Eighteen unselected, consecutive women undergoing IVF were included. Serum and pooled FF samples were obtained simultaneously during routine IVF procedures. Vanin-1 levels were measured by using commercially available ELISA kits. As most of the values were below 0.6 ng/mL, the data are given as optical density. It was found that vanin-1 can be detected in FF and that it is not significantly related to its maternal serum levels (*p* = 0.06). FF vanin-1 levels proved to be higher in non-pregnant as compared to pregnant women (*p* < 0.04). There are significant positive relationships between the FF to serum vanin-1 ratio and body mass index (BMI, *p* < 0.02), anti-Müllerian hormone (AMH, *p* < 0.02) and baseline serum estradiol (*p* < 0.01). Moreover, the FF/serum vanin-1 ratio tended to increase with cumulative FSH dose, but this increase did not reach statistical significance (*p* = 0.064). It may be concluded that FF vanin-1 may serve as a biomarker to predict IVF outcome. To confirm this contention, further studies are to be performed.

## 1. Introduction

During recent decades, great efforts have been made to identify non-invasive molecular markers to assess oocyte competence in women using assisted reproductive technologies. Follicular fluid (FF) has been widely used as the source of biomarkers because it serves as an immediate environment for the oocytes and is an essential medium for the cross-talk between oocytes and ovarian somatic cells. It contains constituents derived from the oocytes, granulosa and theca cells, and from circulating serum with the potential to define oocyte quality and to predict in vitro fertilization (IVF) outcome. In order to achieve this goal, various diagnostic approaches have been applied ranging from measurements of individual biomarkers through panels of biomarkers to the most comprehensive multi-omic techniques. The latter includes genome, transcriptome, proteome and/or metabolome analyses alone or in combination [1,2,3,4,5,6,7].

Indirect evidence is available indicating that members of the vanin family may have an impact on folliculogenesis, oocyte maturation, fertilization and early embryonic development [8,9,10,11]. VNN-1 and VNN-2 genes encode proteins with pantetheinase activity, whereas the truncated protein encoded by VNN-3 has no enzyme activity. Vanin-1 is an epithelial glycosylphosphatidylinositol (GPI)-anchored ectoenzyme that catalyzes the conversion of the pantheteine into pantothenic acid (vitamin B5) and cysteamine. As pantothenic acid is an important precursor of coenzyme A (CoA), its formation is essential to meet CoA’s need for intermediary metabolism. Cysteamine, the other product of pantetheinase either directly or indirectly via the cystamine–glutathione pathway, as antioxidants contribute to the control of cellular redox state.

Vanins are ubiquitously expressed at both mRNA and protein levels with great dominance in the liver, intestine and the kidney [12,13,14,15,16]. There are scant data on their expression in the mammalian reproductive system, specifically in the ovaries and testis [8,9,10,11,17,18,19].

In this preliminary study, attempts were made to reveal whether a/FF in women undergoing IVF contains vanin-1 in assessable amounts by using conventional ELISA kits; b/FF vanin-1 is produced locally by ovarian cells or also originates from the circulating plasma, and c/FF vanin-1 has the potential to predict oocyte competence and fertilization success.

## 2. Materials and Methods

A total of 18 consecutive, non-selected patients undergoing IVF procedures were included in this observational study conducted at the Assisted Reproduction Unit, Department of Obstetrics and Gynecology, University of Pécs, Hungary, between 8 and 22 January 2024 (Table 1).

### 2.1. Controlled Ovarian Hyperstimulation

The study began with routine examinations, including a cervical smear, hormone measurements (FSH, LH, prolactin, estradiol, progesterone, testosterone and TSH) on day 3 and day 21 of the unstimulated cycle, screening for HIV and hepatitis B, hysteroscopy or HyCoSy, and andrological examination. The GnRH antagonist cetrorelix was used in superovulation protocols. Follicular stimulation was achieved with individualized doses of rFSH (150–250 IU daily), adjusted for BMI and age, with up to 300 IU for patients with a previous low response. Supplemental rLH or hMG was administered as needed. Ultrasound assessments were performed every two days beginning on day six of the cycle. Final follicular maturation was induced with 250 µg (6500 IU) recombinant hCG when at least two follicles measured more than 17 mm. Oocyte retrieval was performed 36 h later by ultrasound-guided transvaginal aspiration under sedation.

### 2.2. Embryological Procedures, Embryo Transfer and Clinical Outcomes

Oocytes were collected in G-MOPS™ medium and procedures were performed using Vitrolife “G” series media (Göteborg, Sweden). Fertilization was performed by either intracytoplasmic sperm injection (ICSI) or conventional IVF, depending on sperm parameters (such as low concentration, motility or elevated DNA fragmentation index), maternal age (>35) and previous IVF cycles (>2). For ICSI, mature oocytes (metaphase II) with the first polar body present were selected for injection after hyaluronidase denudation. Fertilization was performed 2–3 h after oocyte retrieval in G-MOPS™ medium. Conventional IVF was performed using bicarbonate-buffered medium (G-IVF™), and successful fertilization was confirmed by the presence of two pronuclei (PN2) in GTL™ medium 24 h later. Fertilization rates were calculated based on the number of oocytes with two pronuclei relative to the total number of fertilized oocytes. Embryo transfer was performed on day 5 after single-step culture in GTL™ medium under controlled atmospheric conditions due to the pre-mixed gases used (5% O_2_, 6% CO_2_, 89% N_2_). At the patients’ request, a maximum of two embryos were transferred, all at the blastocyst stage (day 5); luteal support was provided by daily administration of 400–800 mg progesterone; and clinical pregnancy was determined by serum beta-hCG levels 11–14 days after transfer and confirmed by ultrasound detection of the gestational sac 21 days after transfer.

### 2.3. Sample Processing and Measurement

FF samples were collected during oocyte retrieval, with FF from individual follicles pooled. The samples were immediately centrifuged at 6700× *g* for 10 min at room temperature to remove erythrocytes, leukocytes and granulosa cells. The resulting supernatant was collected and stored at −80 °C for further analysis. Vanin-1 was tested by a sandwich enzyme-linked immunosorbent assay (Human Vanin-1 ELISA kit; Abbexa Ltd., Cambridge, UK; Catalog No: abx153455). Briefly, anti-vanin-1 antibody was coated onto an ELISA plate, and captured vanin-1 from samples was detected using a horseradish peroxidase-conjugated reagent. The conversion of tetramethylbenzidine substrate was proportional to the amount of vanin-1 and was measured as optical density (OD) at 450 nm on a microplate reader.

### 2.4. Statistical Analysis

All statistical analyses were performed with R statistical software ver. 4.41 [20]. Nonparametric methods were used for statistical analysis after a Shapiro–Wilk test for normality. Spearman rank correlation was used to assess the relationship between serum and FF vanin-1 levels. The vanin-1 OD ratio (FF/serum) was calculated as our data approached the lower detection range of the ELISA kit. The Mann–Whitney U test was used to compare vanin-1 levels in serum and FF according to pregnancy status. Factors influencing the vanin-1 OD ratio were analyzed using a generalized linear model (GLM) with inverse Gaussian distribution and log-link function, appropriate for heteroscedastic and right-skewed data. Multicollinearity was tested prior to modeling and no significant strong correlations (R ≥ 0.7) were found between any of the predictor variables involved (online supplementary material, Table S1). Differences were considered significant at *p* < 0.05.

## 3. Results

Vanin-1 was detected in all FF samples, indicating its potential relevance in the reproductive process. Correlation analysis revealed a correlation coefficient of 0.455 between vanin-1 levels in serum and FF, but this was only marginally significant (*p* = 0.06). Vanin-1 optical density (OD) values were measured in both serum (SE) and FF in all patients and by pregnancy outcome. A significant difference was found between vanin-1 levels between the two fluid compartments (FF and serum) (*p* = 0.04), but no significant difference was observed in pregnant women (*p* = 0.48). However, in non-pregnant women the comparison approached significance (*p* = 0.06) (Table 2).

**Figure 1:**
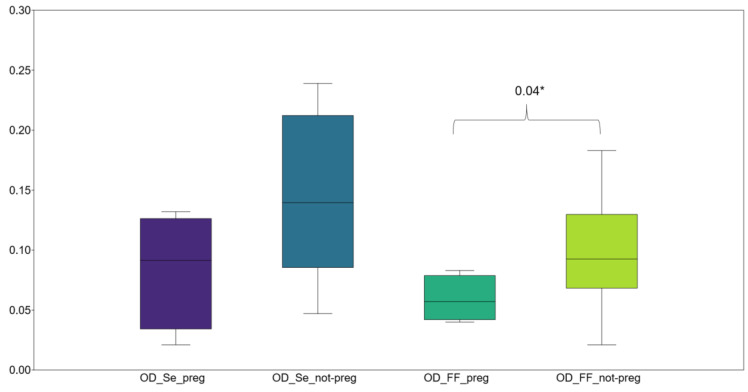
Comparison of the serum and FF OD values based on pregnancy status (OD: optical density; Se: serum; FF: follicular fluid. Preg: pregnant; not-preg: not pregnant). *: *p* < 0.05.

When comparing vanin-1 OD values by pregnancy status, no statistically significant difference in serum levels was found between pregnant and non-pregnant women (*p* = 0.10). However, a significant difference in FF vanin-1 levels was observed between pregnant and non-pregnant women (*p* = 0.04) (Figure 1). The ratio of FF to serum vanin-1 OD (FF/SE) did not show a significant difference between the two groups (*p* = 0.64) (Table 3).

A generalized linear model was used to assess the influence of different factors on the vanin-1 FF to serum OD ratio. The significant predictors in the model included AMH (*p* = 0.0282), BMI (*p* = 0.0237), age (*p* = 0.0210) and estradiol (*p* = 0.0170), indicating that higher AMH, BMI and estradiol levels, as well as age, are associated with an increased vanin-1 ratio. The FSH dose during stimulation was close to significance (*p* = 0.0644). Other variables, such as baseline FSH (*p* = 0.6384) and early-cycle estradiol (*p* = 0.1514), were not significant (Table 4).

## 4. Discussion

Our preliminary study has demonstrated that vanin-1 can be detected in FF by using commercially available ELISA kits and that it is derived at least in part from ovarian cells. Furthermore, FF vanin-1 has the potential to assess oocyte competence and IVF outcome as shown by its elevated levels in women who failed to become pregnant compared with those who became pregnant and by the positive associations of the FF and serum vanin-1 ration with BMI, AMH and baseline estradiol. FSH dose during stimulation fell just short of statistical significance, while baseline FSH and stimulated estradiol proved to be independent of vanin-1

Pitari et al. and Berruyer et al. have reported that vanin-1 serves as a tissue sensor for oxidative stress as the VNNI promoter region contains antioxidant-response-like elements and sites responsible for vanin-1 up-regulation in tissues are exposed to oxidative stress. The enhanced vanin-1 production via the cysteamine–cystamine–ϒ–glutamylcysteine synthase (ϒ-GCS)–glutathione (GSH) pathway reduces thiol groups (-SH) and reversibly generates glutathione disulfide (GSSG). The predominance of GSSG over GSH indicates the reduced capacity of cellular antioxidant defense and intensifies oxidative stress and the related inflammatory reactions.

In support of this concept, convincing evidence has been provided showing that vanin-1-deficient mice exhibit GSH-mediated tissue resistance to oxidative stress. In response to oxidative insult, vanin-1-mutant mice fail to produce a detectable amount of cysteamine, while ϒ-GCS activity is markedly increased with subsequent elevation of tissue GSH [14,21]. The complexity of metabolic pathways influenced by vanin activity is illustrated by Kaskow et al. [15].

Importantly, the VNN1 promoter also contains peroxisome-proliferator-activated receptor-α (PPAR-α) responsive element, and vanin-1 expression can be induced by PPAR-α agonists [22]. PPAR-α-dependent vanin-1 up-regulation has been claimed to be involved in fasting-related lipid mobilization from the tissue fat stores [23]. Pharmacological vanin-1 inhibitors, therefore, may have the potential to control clinically relevant adverse effects of vanin-1 [24]. It is to be emphasized that VNN1 has been shown to regulate PPAR-ϒ mRNA expression, which regulates energy metabolism, and has an important role in the control of innate and adaptive immune responses [25,26].

With respect to the critical role of vanins in oxidative stress, inflammation, cell migration and in metabolic pathways, it appeared to be important to reveal their involvement in female reproduction. Sayasith et al. have investigated the expression, regulation and promoter activation of vanin-2 in bovine follicles prior to ovulation. They analyzed mRNA expression of vanin-2 in granulosa cells obtained at different phases of the estrous cycle and revealed its low or undetectable levels in small and dominant follicles and in the corpus luteum, but it proved to be markedly elevated in ovulatory follicles, suggesting gonadotropin-dependent regulation of VNN2 mRNA in the in vivo model. To confirm this possibility, they performed an in vitro study of granulosa cells of preovulatory follicles obtained 0–24 h post-hCG. It was clearly shown that during the period of 12–24 h post-hCG the vanin-2 mRNA was up-regulated. A similar pattern of vanin-2 transcript expression was seen in their model when granulosa cells were cultured in the presence of forskolin [9]. These observations seem to be in line with our findings that FF vanin-1 levels tended to be related to the FSH dose applied during ovarium stimulation.

The association between the gene expression profile of granulosa cells and oocyte competence was investigated during terminal folliculogenesis in bovines. In the post-FSH withdrawal period of 92 h, three phases could be differentiated according to the developmental competence of oocytes: 20–44 h increasing, 44–68 h optimal plateau and 68–92 h decreasing periods of oocyte competence. Among other genes, VAN1 and NRP1 transcript expression increases with time and follicle size to establish optimal oocyte competence, but the expression of these genes progressively increases further in phase 3 in spite of the decline of the oocyte competence. This implies their involvement in oxidative stress, inflammation and apoptosis, which prevail in this period [17].

In search of metabolic signatures in human FF and to identify predictor(s) of follicular development, Yang J et al. used multi-omic integration technology. They found that compared to large follicles the expression of VNNs, TAS2R and OLFR was markedly suppressed and correlated to each other in small follicles, indicating their critical role in oocyte maturation and fertilization [6].

In patients undergoing IVF, oxidative stress and low-grade inflammation are encountered and may have a critical pathogenetic role [3,27,28,29]. In our study, BMI and AMH were significantly related to FF vanin-1; therefore, it is tempting to postulate that vanin-1 either initiates or intensifies processes that may compromise fertilization.

In conclusion, in spite of the limitations of our preliminary study (the small number of patients, heterogeneous infertility diagnoses and the relatively low sensitivity of ELISA) it provides evidence that FF vanin-1 may serve as a non-invasive, clinically relevant biomarker to predict IVF outcomes. Further studies are needed to explore in more detail the involvement of vanin-1 in fertilization in general, and in the IVF-ET process in particular.

## 5. Conclusions

This preliminary study provides evidence that follicular fluid (FF) vanin-1 levels, measured by ELISA, may serve as a potential biomarker for predicting IVF outcomes. The results showed that FF vanin-1 levels were higher in non-pregnant women compared to those who achieved pregnancy and showed significant associations with BMI, AMH and baseline estradiol levels. Although FSH dose showed a trend towards significance, baseline FSH and stimulated estradiol levels were not predictors of vanin-1 ratios. These observations support the hypothesis that vanin-1 plays a role in follicular and reproductive processes, possibly reflecting oxidative stress and inflammatory pathways. While this study highlights the clinical relevance of FF vanin-1, its limitations, including a small sample size and limited assay sensitivity, underscore the need for further research to validate these findings and elucidate the mechanisms linking vanin-1 to oocyte competence and IVF success.

## Figures and Tables

**Table 1:** Baseline clinical characteristics and laboratory data of all patients and by pregnancy outcome [median (min–max)].

Characteristics	All Patients (18)	Pregnant (N = 4)	Not Pregnant (N = 14)	*p*-Value
Age—(yr)	38 (23–45)	37.5 (24–39)	38.5 (23–45)	0.31
BMI—(kg/m^2^)	22.6 (18.1–33.2)	21.8 (19.0–23.2)	22.7 (18.1–33.2)	0.50
Cause of infertility—no.				
Andrological	8	1	7	-
Tubal factor	6	1	5	-
Idiopathic	4	2	2	-
Basal FSH—(U/L)	8.84 (6.05–18.50)	9.29 (6.83–10.88)	8.84 (6.05–18.50)	0.95
Basal estradiol—(pmol/L)	166.10 (1.05–396.00)	142.50 (64.20–168.00)	182.50 (1.05–396.00)	0.27
AMH—(pmol/L)	1.98 (0.24–7.04)	1.84 (0.43–7.04)	2.35 (0.24–3.80)	0.75
Serum estradiol on the 6th day of stimulation—(pmol/L)	1091 (435–4760)	1544 (712–2429)	1068 (435–4760)	0.76
FSH dose during stimulation—(IU)	2400.0 (1500.0–4296.0)	1737.5 (1500.0–2475.0)	2674.0 (1500.0–4296.0)	0.06 °
Number of oocytes	8.0 (3.0–25.0)	9.5 (9.0–25.0)	7.0 (3.0–24.0)	0.15
Number of MII oocytes	6.0 (2.0–17.0)	9.0 (6.0–17.0)	4.5 (2.0–17.0)	0.15
Number of fertilized oocytes	4.5 (0–11.0)	7.0 (5.0–11.0)	2.5 (0–11.0)	0.04 *
Number of blastocysts	1.5 (0–7.0)	5.0 (2.0–7.0)	1.0 (0–5.0)	0.01 **

°: *p* < 0.1; *: *p* < 0.05; **: *p* < 0.01.

**Table 2:** Serum and FF Vanin-1 OD values [median (min–max)] in relation to IVF outcomes.

	OD FF	OD Serum	*p*-Value
All patients (N = 18)	0.09 (0.021–0.18)	0.12 (0.021–0.24)	0.04 *
Pregnant (N = 4)	0.057 (0.04–0.083)	0.09 (0.021–0.132)	0.48
Not-Pregnant (N = 14)	0.092 (0.021–0.183)	0.139 (0.047–0.239)	0.06 °

°: *p* < 0.1; *: *p* < 0.05.

**Table 3:** Comparison of serum and FF vanin-1 OD values [median (min-max)] depending on the IVF outcome.

	Pregnant (N = 4)	Not Pregnant (N = 14)	*p*-Value
OD Serum	0.09 (0.021–0.132)	0.139 (0.047–0.239)	0.10
OD FF	0.057 (0.04–0.083)	0.092 (0.021–0.183)	0.04 *
Ratio of FF to Serum OD (FF/SE)	0.81 (0.44–1.90)	0.71 (0.28–1.70)	0.64

*: *p* < 0.05.

**Table 4:** Factors influencing the ratio of vanin-1 levels measured in FF and blood serum (OD values) analyzed using a generalized linear model (GLM).

Coefficients	Estimate	Std. Error	t-Value	*p*-Value
(Intercept)	−1.6589	1.3333	−1.244	0.259
Age—(yr)	−0.0881	0.0284	−3.103	0.021 *
BMI—(kg/m^2^)	0.1128	0.0375	3.009	0.023 *
Base serum FSH—(U/L)	−0.0150	0.0304	−0.495	0.638
Base serum estradiol—(pmol/L)	0.0044	0.0013	3.273	0.017 *
AMH—(pmol/L)	0.2177	0.0757	2.877	0.028 *
Serum estradiol on the 6th day of stimulation—(pmol/L)	−0.0001	0.0001	−1.644	0.151
FSH dose during stimulation—(IU)	0.0004	0.0002	2.262	0.064 °

°: *p* < 0.1; *: *p* < 0.05.

## Data Availability

The data supporting the findings of this study are available from the corresponding author upon reasonable request.

## Supplementary Materials

The following supporting information can be downloaded at https://www.mdpi.com/article/10.3390/antiox14020133/s1. Table S1: Correlation matrix of factors influencing the follicular fluid/serum vanin-1 ratio.

## Abbreviations

BMI	body mass index
AMH	anti-Müllerian hormone
(r)FSH	(recombinant) follicle stimulating hormone
(r)LH	(recombinant) luteinizing hormone
hMG	human menopausal gonadotropin
