# Supplementary material for: Follicular Fluid Vanin-1 Levels in Patients Undergoing Ivf: A Preliminary Study
Source: Antioxidants (Basel). 2025 Jan 23;14(2):133. doi: 10.3390/antiox14020133 (PMC11851465; doi:10.3390/antiox14020133)
Supplement: Supplementary file 1 [file antioxidants-14-00133-s001.zip › antioxidants-3357930-supplementary.pdf]

Supplementary Table S1. Correlation matrix of factors influencing the follicular fluid/serum Vanin-1 ratio.

Statistic / p-value	Age – (yr)	BMI – (kg/m²)	FSH dose during stimulation – (IU)	Mean of serum estradiol on the 6th day of stimulation – (pmol/l)	Base serum FSH – (U/l)	Base estradiol – (pmol/l)	AMH – (pmol/l)
Age – (yr)		0.34	0.005*	0.16	0.14	0.31	0.41
BMI – (kg/m²)	-0.2348		0.53	0.54	0.78	0.04*	0.37
FSH dose during stimulation – (IU)	0.6341	0.1586		0.06	0.32	0.92	0.09
Serum estradiol on the 6th day of stimulation – (pmol/l)	-0.3509	-0.1616	-0.4668		0.04*	0.41	0.09
Base serum FSH – (U/l)	0.3652	-0.07245	0.2469	-0.4893		0.78	0.38
Base serum estradiol – (pmol/l)	0.2557	-0.4973	0.0223	-0.2140	0.0718		0.29
AMH – (pmol/l)	-0.2319	-0.2469	-0.4738	0.4675	-0.2442	-0.2914	
